# Supplementary material for: Association between Breakfast Skipping and Body Weight—A Systematic Review and Meta-Analysis of Observational Longitudinal Studies
Source: Nutrients. 2021 Jan 19;13(1):272. doi: 10.3390/nu13010272 (PMC7832891; doi:10.3390/nu13010272)
Supplement: Supplementary file 1 [file nutrients-13-00272-s001.pdf]

Supplementary Table S1. Studies excluded by reason.

**Exclusion Criteria (*n* excluded)**	**References**
Study design (*n* = 68)	[1–68]
Exposure (*n* = 30)	[69–98]
Language (*n* = 22)	[99–120]
Outcome (*n* = 21)	[121–141]
Exposure & outcome (*n* = 15)	[142–156]
Target group (*n* = 9)	[157–165]
No effect size (*n* = 3)	[166–168]
Authors not reached (*n* = 2)	[169,170]

Supplementary Table S2. Risk of bias in included studies according to ROBINS-I.

References	Risk of Bias Domains							Overall Risk of Bias
	Confounding	Selection	Exposure classification	Misclassification	Missing data	Outcome measurement	Reporting	
Goto et al., 2010 [171]								serious risk
Guinter et al., 2020 [172]								moderate risk
Hurst and Fukuda, 2018 [173]								critical risk
Kahleova et al., 2017 [174]								serious risk
Kito et al., 2019 [175]								serious risk
Nooyens et al., 2005 [176]								serious risk
Odegaard et al., 2013 [177]								moderate risk
Smith et al., 2017 [178]								serious risk
van der Heijden et al., 2007 [179]								serious risk

Legend: no information on risk of bias (grey); low risk of bias (green); moderate risk of bias (yellow); serious risk of bias (orange); critical risk of bias (red). ROBINS-I = Risk of Bias in Non-randomized Studies of Interventions.

Supplementary Table S3. Quality of evidence of meta-analyses referring to NutriGRADE.

Meta-analysis (*n* studies)	Quality Assessment								Summary of Findings		Meta-Evidence
	Risk of bias	Precision	Heterogeneity	Directness	Publication bias	Funding bias	Effect size	Dose-response	Participants	ES (95% CI)	
	(0–2 *p*)	(0–1 *p*)	(0–1.6 *p*)	(0–1 *p*)	(0–1 *p*)	(0–1 *p*)	(0–1 *p*)	(0–1 *p*)	*n cases* *n controls*		(∑ 0–8.6 P)
Overweight (*n* = 2)	1	1	0.4	0	0	1	0	0	25,764 79,487	RR = 1.11 (1.04, 1.19)	very low (∑ 3.4)
BMI change (*n* = 2)	1	0	0.2	0	0	1	0	0	108,413*	β = -0.02 (−0.05, 0.01)	very low (∑ 2.2)

Legend: NutriGRADE = Nutrition Grading of Recommendations Assessment, Development and Evaluation; BMI = body mass index; *n participants; no information on cases and controls; ES = effect estimate; CI = confidence interval.

References

1. Al-Rethaiaa, A.S.; Fahmy, A.-E.A.; Al-Shwaiyat, N.M. Obesity and eating habits among college students in Saudi Arabia: a cross sectional study. *Nutr. J.* **2010**, *9*, 39, doi:10.1186/1475-2891-9-39.
2. Aparicio, A.; Rodriguez-Rodriguez, E.E.; Aranceta-Bartrina, J.; Gil, A.; Gonzalez-Gross, M.; Serra-Majem, L.; Varela-Moreiras, G.; Ortega, R.M. Differences in meal patterns and timing with regard to central obesity in the ANIBES ('Anthropometric data, macronutrients and micronutrients intake, practice of physical activity, socioeconomic data and lifestyles in Spain') Study. *Public Health Nutr.* **2017**, *20*, 2364–2373, doi:10.1017/S1368980017000635.
3. Azadbakht, L.; Haghighatdoost, F.; Feizi, A.; Esmaillzadeh, A. Breakfast eating pattern and its association with dietary quality indices and anthropometric measurements in young women in Isfahan. *Nutrition* **2013**, *29*, 420–425, doi:10.1016/j.nut.2012.07.008.
4. Barnes, A.S.; Kimbro, R.T. Descriptive study of educated African American women successful at weight-loss maintenance through lifestyle changes. *J. Gen. Intern. Med.* **2012**, *27*, 1272–1279, doi:10.1007/s11606-012-2060-2.
5. Barr, S.I.; DiFrancesco, L.; Fulgoni, V.L.3. Association of breakfast consumption with body mass index and prevalence of overweight/obesity in a nationally-representative survey of Canadian adults. *Nutr. J.* **2016**, *15*, 33, doi:10.1186/s12937-016-0151-3.
6. Batista-Jorge, G.C.; Barcala-Jorge, A.S.; Oliveira Dias, A.F.; Silveira, M.F.; Farias Lelis, D. de; Oliveira Andrade, J.M.; Claro, R.M.; de Paula, Alfredo Mauricio Batista; Guimaraes, A.L.S.; Ferreira, A.V.; et al. Nutritional Status Associated to Skipping Breakfast in Brazilian Health Service Patients. *Ann. Nutr. Metab.* **2016**, *69*, 31–40, doi:10.1159/000447363.
7. Berg, C.; Lappas, G.; Wolk, A.; Strandhagen, E.; Toren, K.; Rosengren, A.; Thelle, D.; Lissner, L. Eating patterns and portion size associated with obesity in a Swedish population. *Appetite* **2009**, *52*, 21–26, doi:10.1016/j.appet.2008.07.008.
8. Bjornara, H.B.; Vik, F.N.; Brug, J.; Manios, Y.; Bourdeaudhuij, I. de; Jan, N.; Maes, L.; Moreno, L.A.; Dossegger, A.; Bere, E. The association of breakfast skipping and television viewing at breakfast with weight status among parents of 10-12-year-olds in eight European countries; the ENERGY (EuropeaN Energy balance Research to prevent excessive weight Gain among Youth) cross-sectional study. *Public Health Nutr.* **2014**, *17*, 906–914, doi:10.1017/S136898001300061X.
9. Boo, N.Y.; Chia, G.J.Q.; Wong, L.C.; Chew, R.M.; Chong, W.; Loo, R.C.N. The prevalence of obesity among clinical students in a Malaysian medical school. *Singapore Med. J.* **2010**, *51*, 126–132.
10. Brikou, D.; Zannidi, D.; Karfopoulou, E.; Anastasiou, C.A.; Yannakoulia, M. Breakfast consumption and weight-loss maintenance: results from the MedWeight study. *Br. J. Nutr.* **2016**, *115*, 2246–2251, doi:10.1017/S0007114516001550.
11. Carels, R.A.; Young, K.M.; Coit, C.; Clayton, A.M.; Spencer, A.; Wagner, M. Skipping meals and alcohol consumption. The regulation of energy intake and expenditure among weight loss participants. *Appetite* **2008**, *51*, 538–545, doi:10.1016/j.appet.2008.04.006.
12. Chatelan, A.; Castetbon, K.; Pasquier, J.; Allemann, C.; Zuber, A.; Camenzind-Frey, E.; Zuberbuehler, C.A.; Bochud, M. Association between breakfast composition and abdominal obesity in the Swiss adult population eating breakfast regularly. *Int. J. Behav. Nutr. Phys. Act.* **2018**, *15*, doi:10.1186/s12966-018-0752-7.
13. Cho, S.; Dietrich, M.; Brown, C.J.P.; Clark, C.A.; Block, G. The effect of breakfast type on total daily energy intake and body mass index: results from the Third National Health and Nutrition Examination Survey (NHANES III). *J. Am. Coll. Nutr.* **2003**, *22*, 296–302.
14. Chung, H.Y.; Song, M.K.; Park, M.H. A Study of the Anthropometric Indices and Eating Habits of Female College Students. *J Community Nutrition* **2003**, *5*, 21–28.
15. Chung, S.-J.; Lee, Y.; Lee, S.; Choi, K. Breakfast skipping and breakfast type are associated with daily nutrient intakes and metabolic syndrome in Korean adults. *Nutr. Res. Pract.* **2015**, *9*, 288–295, doi:10.4162/nrp.2015.9.3.288.
16. Deshmukh-Taskar, P.; Nicklas, T.A.; Radcliffe, J.D.; O'Neil, C.E.; Liu, Y. The relationship of breakfast skipping and type of breakfast consumed with overweight/obesity, abdominal obesity, other cardiometabolic risk factors and the metabolic syndrome in young adults. The National Health and Nutrition Examination Survey (NHANES): 1999-2006. *Public Health Nutr.* **2013**, *16*, 2073–2082, doi:10.1017/S1368980012004296.
17. Di Giuseppe, R.; Di Castelnuovo, A.; Melegari, C.; Lucia, F. de; Santimone, I.; Sciarretta, A.; Barisciano, P.; Persichillo, M.; Curtis, A. de; Zito, F.; et al. Typical breakfast food consumption and risk factors for cardiovascular disease in a large sample of Italian adults. *Nutr. Metab. Cardiovasc. Dis.* **2012**, *22*, 347–354, doi:10.1016/j.numecd.2010.07.006.
18. Fayet-Moore, F.; McConnell, A.; Cassettari, T.; Petocz, P. Breakfast Choice Is Associated with Nutrient, Food Group and Discretionary Intakes in Australian Adults at Both Breakfast and the Rest of the Day. *Nutrients* **2019**, *11*, doi:10.3390/nu11010175.

19. Forslund, H.B.; Lindroos, A.K.; Sjöström, L.; Lissner, L. Meal patterns and obesity in Swedish women-a simple instrument describing usual meal types, frequency and temporal distribution. *Eur. J. Clin. Nutr.* **2002**, *56*, 740–747, doi:10.1038/sj.ejcn.1601387.
20. Fuglestad, P.T.; Jeffery, R.W.; Sherwood, N.E. Lifestyle patterns associated with diet, physical activity, body mass index and amount of recent weight loss in a sample of successful weight losers. *Int. J. Behav. Nutr. Phys. Act.* **2012**, *9*, 79, doi:10.1186/1479-5868-9-79.
21. Fujiwara, T. Skipping breakfast is associated with dysmenorrhea in young women in Japan. *Int. J. Food Sci. Nutr.* **2003**, *54*, 505–509, doi:10.1080/09637480310001622369.
22. Fujiwara, T.; Nakata, R. Skipping breakfast is associated with reproductive dysfunction in post-adolescent female college students. *Appetite* **2010**, *55*, 714–717, doi:10.1016/j.appet.2010.08.005.
23. Fujiwara, T.; Sato, N.; Awaji, H.; Sakamoto, H.; Nakata, R. Skipping breakfast adversely affects menstrual disorders in young college students. *Int. J. Food Sci. Nutr.* **2009**, *60*, 23–31, doi:10.1080/09637480802260998.
24. Gazibara, T.; Kisic Tepavcevic, D.B.; Popovic, A.; Pekmezovic, T. Eating habits and body-weights of students of the university of belgrade, serbia: a cross-sectional study. *J. Health Popul. Nutr.* **2013**, *31*, 330–333.
25. Gouda, M.; Matsukawa, M.; Iijima, H. Associations between eating habits and glycemic control and obesity in Japanese workers with type 2 diabetes mellitus. *Diabetes Metab. Syndr. Obes.* **2018**, *11*, 647–658, doi:10.2147/DMSO.S176749.
26. Grujic, V.; Cvejin, M.M.; Nikolic, E.A.; Dragnic, N.; Jovanovic, V.M.; Kvrgic, S.; Travar, S. Association between obesity and socioeconomic factors and lifestyle. *Vojnosanit. Pregl.* **2009**, *66*, 705–710.
27. Hassan, N.E.; Wahba, S.A.; El-Masry, S.A.; Elhamid, E.R.A.; Boseila, S.A.W.; Ahmed, N.H.; Ibrahim, T.S. Eating Habits and Lifestyles among a Sample of Obese Working Egyptian Women. *Open Access Maced. J. Med. Sci.* **2015**, *3*, 12–17, doi:10.3889/oamjms.2015.005.
28. Heinrich, K.M.; Maddock, J. Multiple health behaviors in an ethnically diverse sample of adults with risk factors for cardiovascular disease. *Perm. J.* **2011**, *15*, 12–18.
29. Hingorjo, M.R.; Syed, S.; Qureshi, M.A. Overweight and obesity in students of a dental college of Karachi: lifestyle influence and measurement by an appropriate anthropometric index. *J. Pak. Med. Assoc.* **2009**, *59*, 528–532.
30. Huang, C.-J.; Hu, H.-T.; Fan, Y.-C.; Liao, Y.-M.; Tsai, P.-S. Associations of breakfast skipping with obesity and health-related quality of life: evidence from a national survey in Taiwan. *Int. J. Obes. (Lond)* **2010**, *34*, 720–725, doi:10.1038/ijo.2009.285.
31. Jang, H.J.; Kim, B.S.; Won, C.W.; Kim, S.Y.; Seo, M.W. The Relationship between Psychological Factors and Weight Gain. *Korean J. Fam. Med.* **2020**, doi:10.4082/kjfm.19.0049.
32. Kant, A.K.; Andon, M.B.; Angelopoulos, T.J.; Rippe, J.M. Association of breakfast energy density with diet quality and body mass index in American adults: National Health and Nutrition Examination Surveys, 1999-2004. *Am. J. Clin. Nutr.* **2008**, *88*, 1396–1404, doi:10.3945/ajcn.2008.26171.
33. Kent, L.M.; Worsley, A. Breakfast size is related to body mass index for men, but not women. *Nutr. Res.* **2010**, *30*, 240–245, doi:10.1016/j.nutres.2010.03.006.
34. Keski-Rahkonen, A.; Kaprio, J.; Rissanen, A.; Virkkunen, M.; Rose, R.J. Breakfast skipping and health-compromising behaviors in adolescents and adults. *Eur. J. Clin. Nutr.* **2003**, *57*, 842–853, doi:10.1038/sj.ejcn.1601618.
35. Ko, M.-S. The comparison in daily intake of nutrients, dietary habits and body composition of female college students by body mass index. *Nutr. Res. Pract.* **2007**, *1*, 131–142, doi:10.4162/nrp.2007.1.2.131.
36. Kong, A.; Beresford, S.A.A.; Alfano, C.M.; Foster-Schubert, K.E.; Neuhouser, M.L.; Johnson, D.B.; Duggan, C.; Wang, C.-Y.; Xiao, L.; Jeffery, R.W.; et al. Self-monitoring and eating-related behaviors are associated with 12-month weight loss in postmenopausal overweight-to-obese women. *J. Acad. Nutr. Diet.* **2012**, *112*, 1428–1435, doi:10.1016/j.jand.2012.05.014.
37. Kutsuma, A.; Nakajima, K.; Suwa, K. Potential Association between Breakfast Skipping and Concomitant Late-Night-Dinner Eating with Metabolic Syndrome and Proteinuria in the Japanese Population. *Scientifica (Cairo)* **2014**, *2014*, 253581, doi:10.1155/2014/253581.
38. Lee, J.S.; Mishra, G.; Hayashi, K.; Watanabe, E.; Mori, K.; Kawakubo, K. Combined eating behaviors and overweight: Eating quickly, late evening meals, and skipping breakfast. *Eat. Behav.* **2016**, *21*, 84–88, doi:10.1016/j.eatbeh.2016.01.009.
39. Lee, S.-K. Acculturation, meal frequency, eating-out, and body weight in Korean Americans. *Nutr. Res. Pract.* **2008**, *2*, 269–274, doi:10.4162/nrp.2008.2.4.269.
40. Ma, Y.; Bertone, E.R.; Stanek, E.J.3.; Reed, G.W.; Hebert, J.R.; Cohen, N.L.; Merriam, P.A.; Ockene, I.S. Association between eating patterns and obesity in a free-living US adult population. *Am. J. Epidemiol.* **2003**, *158*, 85–92.
41. Maksimović, M.Ž.; Gudelj Rakić, J.M.; Vlajinac, H.D.; Vasiljević, N.D.; Marinković, J.M. Relationship between health behaviour and body mass index in the Serbian adult population: data from National Health Survey 2013. *Int. J. Public Health* **2016**, *61*, 57–68, doi:10.1007/s00038-015-0765-9.

42. Malinauskas, B.M.; Raedeke, T.D.; Aeby, V.G.; Smith, J.L.; Dallas, M.B. Dieting practices, weight perceptions, and body composition: a comparison of normal weight, overweight, and obese college females. *Nutr. J.* **2006**, *5*, 11, doi:10.1186/1475-2891-5-11.
43. Mansouri, M.; Hasani-Ranjbar, S.; Yaghubi, H.; Rahmani, J.; Tabrizi, Y.M.; Keshtkar, A.; Varmaghani, M.; Sharifi, F.; Sadeghi, O. Breakfast consumption pattern and its association with overweight and obesity among university students: a population-based study. *Eat. Weight Disord.* **2018**, doi:10.1007/s40519-018-0609-8.
44. Marin-Guerrero, A.C.; Gutierrez-Fisac, J.L.; Guallar-Castillon, P.; Banegas, J.R.; Rodriguez-Artalejo, F. Eating behaviours and obesity in the adult population of Spain. *Br. J. Nutr.* **2008**, *100*, 1142–1148, doi:10.1017/S0007114508966137.
45. Masheb, R.M.; Grilo, C.M. Eating patterns and breakfast consumption in obese patients with binge eating disorder. *Behav. Res. Ther.* **2006**, *44*, 1545–1553, doi:10.1016/j.brat.2005.10.013.
46. Masheb, R.M.; Grilo, C.M.; White, M.A. An examination of eating patterns in community women with bulimia nervosa and binge eating disorder. *Int. J. Eat. Disord.* **2011**, *44*, 618–624, doi:10.1002/eat.20853.
47. Maugeri, A.; Kunzova, S.; Medina-Inojosa, J.R.; Agodi, A.; Barchitta, M.; Homolka, M.; Kiacova, N.; Bauerova, H.; Sochor, O.; Lopez-Jimenez, F.; et al. Association between eating time interval and frequency with ideal cardiovascular health: Results from a random sample Czech urban population. *Nutr. Metab. Cardiovasc. Dis.* **2018**, doi:10.1016/j.numecd.2018.04.002.
48. McCrory, M.A.; Behrens, B.A.; Malkoc, K.S.; Campbell, W.W.; Boushey, C.J. Is Breakfast or Breakfast Skipping Associated with Adiposity in Adults? Methodological Considerations of the Breakfast Definition. *Obesity (Silver Spring)* **2009**, *17*, S133.
49. Mills, J.P.; Perry, C.D.; Reicks, M. Eating frequency is associated with energy intake but not obesity in midlife women. *Obesity (Silver Spring)* **2011**, *19*, 552–559, doi:10.1038/oby.2010.265.
50. Min, C.; Noh, H.; Kang, Y.-S.; Sim, H.J.; Baik, H.W.; Song, W.O.; Yoon, J.; Park, Y.-H.; Joung, H. Skipping breakfast is associated with diet quality and metabolic syndrome risk factors of adults. *Nutr. Res. Pract.* **2011**, *5*, 455–463, doi:10.4162/nrp.2011.5.5.455.
51. Mostad, I.L.; Langaas, M.; Grill, V. Central obesity is associated with lower intake of whole-grain bread and less frequent breakfast and lunch: results from the HUNT study, an adult all-population survey. *Appl. Physiol. Nutr. Metab.* **2014**, *39*, 819–828, doi:10.1139/apnm-2013-0356.
52. Musaiger, A.O.; Radwan, H.M. Social and dietary factors associated with obesity in university female students in United Arab Emirates. *J. R. Soc. Health* **1995**, *115*, 96–99.
53. Navarro-Gonzalez, I.; Lopez-Nicolas, R.; Rodriguez-Tadeo, A.; Ros-Berruezo, G.; Martinez-Marin, M.; Domenech-Asensi, G. Adherence to the Mediterranean diet by nursing students of Murcia (Spain). *Nutr. Hosp.* **2014**, *30*, 165–172, doi:10.3305/nh.2014.30.1.7413.
54. Navia, B.; Lopez-Sobaler, A.M.; Villalobos, T.; Aranceta-Bartrina, J.; Gil, A.; Gonzalez-Gross, M.; Serra-Majem, L.; Varela-Moreiras, G.; Ortega, R.M. Breakfast habits and differences regarding abdominal obesity in a cross-sectional study in Spanish adults: The ANIBES study. *PLoS One* **2017**, *12*, doi:10.1371/journal.pone.0188828.
55. Olafsdottir, A.S.; Torfadottir, J.E.; Arngrimsson, S.A. Health Behavior and Metabolic Risk Factors Associated with Normal Weight Obesity in Adolescents. *PLoS One* **2016**, *11*, e0161451, doi:10.1371/journal.pone.0161451.
56. O'Neil, C.E.; Nicklas, T.A.; Fulgoni, V.L. Nutrient intake, diet quality, and weight/adiposity parameters in breakfast patterns compared with no breakfast in adults: National Health and Nutrition Examination Survey 2001-2008. *J. Acad. Nutr. Diet.* **2014**, *114*, S27-43, doi:10.1016/j.jand.2014.08.021.
57. Otaki, N.; Obayashi, K.; Saeki, K.; Kitagawa, M.; Tone, N.; Kurumatani, N. Relationship between Breakfast Skipping and Obesity among Elderly: Cross-Sectional Analysis of the HEIJO-KYO Study. *J. Nutr. Health Aging* **2017**, *21*, 501–504, doi:10.1007/s12603-016-0792-0.
58. Pot, G.K.; Hardy, R.; Stephen, A.M. Irregular consumption of energy intake in meals is associated with a higher cardiometabolic risk in adults of a British birth cohort. *Int. J. Obes. (Lond)* **2014**, *38*, 1518–1524, doi:10.1038/ijo.2014.51.
59. Raynor, H.A.; Jeffery, R.W.; Ruggiero, A.M.; Clark, J.M.; Delahanty, L.M. Weight loss strategies associated with BMI in overweight adults with type 2 diabetes at entry into the Look AHEAD (Action for Health in Diabetes) trial. *Diabetes Care* **2008**, *31*, 1299–1304, doi:10.2337/dc07-2295.
60. Reeves, S.; Halsey, L.G.; McMeel, Y.; Huber, J.W. Breakfast habits, beliefs and measures of health and wellbeing in a nationally representative UK sample. *Appetite* **2013**, *60*, 51–57, doi:10.1016/j.appet.2012.09.024.
61. Smith, K.J.; McNaughton, S.A.; Cleland, V.J.; Crawford, D.; Ball, K. Health, Behavioral, Cognitive, and Social Correlates of Breakfast Skipping among Women Living in Socioeconomically Disadvantaged Neighborhoods. *J. Nutr.* **2013**, *143*, 1774–1784, doi:10.3945/jn.113.181396.
62. Song, W.O.; Chun, O.K.; Obayashi, S.; Cho, S.; Chung, C.E. Is consumption of breakfast associated with body mass index in US adults? *J. Am. Diet. Assoc.* **2005**, *105*, 1373–1382, doi:10.1016/j.jada.2005.06.002.
63. Watanabe, Y.; Saito, I.; Henmi, I.; Yoshimura, K.; Maruyama, K.; Yamauchi, K.; Matsuo, T.; Kato, T.; Tanigawa, T.; Kishida, T.; et al. Skipping Breakfast is Correlated with Obesity. *J. Rural Med.* **2014**, *9*, 51–58, doi:10.2185/jrm.2887.

64. Wright, M.; Adair, L.; James, C.; Amuleru-Marshall, O.; Peltzer, K.; Pengpid, S.; Samuels, T.A. The association of nutrition behaviors and physical activity with general and central obesity in Caribbean undergraduate students. *Rev. Panam. Salud Publica* **2015**, *38*, 278–285.
65. Wyatt, H.R.; Grunwald, G.K.; Mosca, C.L.; Klem, M.L.; Wing, R.R.; Hill, J.O. Long-term weight loss and breakfast in subjects in the National Weight Control Registry. *Obes. Res.* **2002**, *10*, 78–82, doi:10.1038/oby.2002.13.
66. Yasuda, J.; Asako, M.; Arimitsu, T.; Fujita, S. Skipping breakfast is associated with lower fat-free mass in healthy young subjects: a cross-sectional study. *Nutr. Res.* **2018**, *60*, 26–32, doi:10.1016/j.nutres.2018.09.006.
67. Zalewska, M.; Maciorkowska, E. Selected nutritional habits of teenagers associated with overweight and obesity. *PeerJ* **2017**, *5*, e3681, doi:10.7717/peerj.3681.
68. Zhang, L.; Cordeiro, L.S.; Liu, J.; Ma, Y. The Association between Breakfast Skipping and Body Weight, Nutrient Intake, and Metabolic Measures among Participants with Metabolic Syndrome. *Nutrients* **2017**, *9*, doi:10.3390/nu9040384.
69. Albertson, A.M.; Goebel, M.T.; Kolberg, L.W.; Crockett, S.J. Breakfast and ready-to-eat cereal consumption habits of adult women in the US population and the relationship with energy intake and body mass index? *Obes. Res.* **2012**, *9*, 183S.
70. Albertson, A.M.; Wold, A.C.; Joshi, N. Ready-to-Eat Cereal Consumption Patterns: The Relationship to Nutrient Intake, Whole Grain Intake, and Body Mass Index in an Older American Population. *J. Aging Res.* **2012**, *2012*, 631310, doi:10.1155/2012/631310.
71. al-Isa, A.N. Obesity among Kuwait University students: an explorative study. *J. R. Soc. Promot. Health* **1999**, *119*, 223–227.
72. Bazzano, L.A.; Song, Y.; Bubes, V.; Good, C.K.; Manson, J.E.; Liu, S. Dietary intake of whole and refined grain breakfast cereals and weight gain in men. *Obes. Res.* **2005**, *13*, 1952–1960, doi:10.1038/oby.2005.240.
73. Beck, K.L.; Jones, B.; Ullah, I.; McNaughton, S.A.; Haslett, S.J.; Stonehouse, W. Associations between dietary patterns, socio-demographic factors and anthropometric measurements in adult New Zealanders: an analysis of data from the 2008/09 New Zealand Adult Nutrition Survey. *Eur. J. Nutr.* **2018**, *57*, 1421–1433, doi:10.1007/s00394-017-1421-3.
74. Berteus Forslund, H.; Torgerson, J.S.; Sjostrom, L.; Lindroos, A.K. Snacking frequency in relation to energy intake and food choices in obese men and women compared to a reference population. *Int. J. Obes. (Lond)* **2005**, *29*, 711–719, doi:10.1038/sj.ijo.0802950.
75. Ericson, U.; Brunkwall, L.; Alves Dias, J.; Drake, I.; Hellstrand, S.; Gullberg, B.; Sonestedt, E.; Nilsson, P.M.; Wirfalt, E.; Orho-Melander, M. Food patterns in relation to weight change and incidence of type 2 diabetes, coronary events and stroke in the Malmo Diet and Cancer cohort. *Eur. J. Nutr.* **2018**, doi:10.1007/s00394-018-1727-9.
76. Esquirol, Y.; Bongard, V.; Mabile, L.; Jonnier, B.; Soulat, J.-M.; Perret, B. Shift work and metabolic syndrome: respective impacts of job strain, physical activity, and dietary rhythms. *Chronobiol. Int.* **2009**, *26*, 544–559, doi:10.1080/07420520902821176.
77. Gittelsohn, J.; Wolever, T.M.; Harris, S.B.; Harris-Giraldo, R.; Hanley, A.J.; Zinman, B. Specific patterns of food consumption and preparation are associated with diabetes and obesity in a Native Canadian community. *J. Nutr.* **1998**, *128*, 541–547, doi:10.1093/jn/128.3.541.
78. Grammatikopoulou, M.G.; Maraki, M.I.; Giannopoulou, D.; Poulimeneas, D.; Sidossis, L.S.; Tsigga, M. Similar Mediterranean diet adherence but greater central adiposity is observed among Greek diaspora adolescents living in Istanbul, compared to Athens. *Ethn. Health* **2018**, *23*, 221–232, doi:10.1080/13557858.2016.1258043.
79. Heerman, W.J.; Jackson, N.; Hargreaves, M.; Mulvaney, S.A.; Schlundt, D.; Wallston, K.A.; Rothman, R.L. Clusters of Healthy and Unhealthy Eating Behaviors Are Associated With Body Mass Index Among Adults. *J. Nutr. Educ. Behav.* **2017**, *49*, 415+, doi:10.1016/j.jneb.2017.02.001.
80. Hermenegildo, Y.; Lopez-Garcia, E.; Garcia-Esquinas, E.; Perez-Tasigchana, R.F.; Rodriguez-Artalejo, F.; Guallar-Castillon, P. Distribution of energy intake throughout the day and weight gain: a population-based cohort study in Spain. *Br. J. Nutr.* **2016**, *115*, 2003–2010, doi:10.1017/S0007114516000891.
81. Kant, A.K.; Schatzkin, A.; Graubard, B.I.; Ballard-Barbash, R. Frequency of eating occasions and weight change in the NHANES I Epidemiologic Follow-up Study. *Int. J. Obes. Relat. Metab. Disord.* **1995**, *19*, 468–474.
82. Kent, L.M.; Worsley, A. Trends in BMI, diet and lifestyle between 1976 and 2005 in North Sydney. *Asia Pac. J. Clin. Nutr.* **2009**, *18*, 453–461.
83. Mouchacca, J.; Abbott, G.R.; Ball, K. Associations between psychological stress, eating, physical activity, sedentary behaviours and body weight among women: a longitudinal study. *BMC Public Health* **2013**, *13*, 828, doi:10.1186/1471-2458-13-828.
84. Ortega, R.M.; Redondo, M.R.; Lopez-Sobaler, A.M.; Quintas, M.E.; Zamora, M.J.; Andres, P.; Encinas-Sotillos, A. Associations between obesity, breakfast-time food habits and intake of energy and nutrients in a group of elderly Madrid residents. *J. Am. Coll. Nutr.* **1996**, *15*, 65–72.
85. Ortega Anta, R.M.; Lopez-Solaber, A.M.; Perez-Farinos, N. Associated factors of obesity in Spanish representative samples. *Nutr. Hosp.* **2013**, *28 Suppl 5*, 56–62, doi:10.3305/nh.2013.28.sup5.6918.

86. Osaka, R.; Nanakorn, S.; Sanseeha, L.; Nagahiro, C.; Kodama, N. Healthy dietary habits, body mass index, and predictors among nursing students, northeast Thailand. *Southeast Asian J. Trop. Med. Public Health* **1999**, *30*, 115–121.
87. Pot, G.K.; Hardy, R.; Stephen, A.M. Irregularity of energy intake at meals: prospective associations with the metabolic syndrome in adults of the 1946 British birth cohort. *Br. J. Nutr.* **2016**, *115*, 315–323, doi:10.1017/S0007114515004407.
88. Purslow, L.R.; Sandhu, M.S.; Forouhi, N.; Young, E.H.; Luben, R.N.; Welch, A.A.; Khaw, K.-T.; Bingham, S.A.; Wareham, N.J. Energy intake at breakfast and weight change: prospective study of 6,764 middle-aged men and women. *Am. J. Epidemiol.* **2008**, *167*, 188–192, doi:10.1093/aje/kwm309.
89. Quatela, A.; Callister, R.; Patterson, A.J.; McEvoy, M.; MacDonald-Wicks, L.K. Breakfast Cereal Consumption and Obesity Risk amongst the Mid-Age Cohort of the Australian Longitudinal Study on Women's Health. *Healthcare (Basel)* **2017**, *5*, doi:10.3390/healthcare5030049.
90. Senekal, M.; Lasker, G.L.; van Velden, L.; Laubscher, R.; Temple, N.J. Weight-loss strategies of South African female university students and comparison of weight management-related characteristics between dieters and non-dieters. *BMC Public Health* **2016**, *16*, 918, doi:10.1186/s12889-016-3576-x.
91. Sinaga, M.; Teshome, M.S.; Kidane, R.; Yemane, T.; Tegene, E.; Lindstrom, D.; Belachew, T. Metabolic Effects of Fasting and Animal Source Food Avoidance in an Ethiopian Adult Cohort. *Sci. Rep.* **2019**, *9*, 16964, doi:10.1038/s41598-019-53185-3.
92. Sjoberg, A.; Barrenas, M.-L.; Brann, E.; Chaplin, J.E.; Dahlgren, J.; Marild, S.; Lissner, L.; Albertsson-Wikland, K. Body size and lifestyle in an urban population entering adulthood: the 'Grow up Gothenburg' study. *Acta Paediatr.* **2012**, *101*, 964–972, doi:10.1111/j.1651-2227.2012.02722.x.
93. Soreca, I.; Wallace, M.L.; Hall, M.H.; Hasler, B.P.; Frank, E.; Kupfer, D.J. The association between meal timing and frequency with cardiometabolic profile in patients with bipolar disorder. *Acta Psychiatr. Scand.* **2016**, *133*, 453–458, doi:10.1111/acps.12578.
94. Suliburska, J.; Bogdański, P.; Pupek-Musialik, D.; Głód-Nawrocka, M.; Krauss, H.; Piątek, J. Analysis of lifestyle of young adults in the rural and urban areas. *Ann. Agric. Environ. Med.* **2012**, *19*, 135–139.
95. Summerbell, C.D.; Moody, R.C.; Shanks, J.; Stock, M.J.; Geissler, C. Relationship between feeding pattern and body mass index in 220 free-living people in four age groups. *Eur. J. Clin. Nutr.* **1996**, *50*, 513–519.
96. Tian, J.; Gall, S.L.; Smith, K.J.; Dwyer, T.; Venn, A.J. Worsening Dietary and Physical Activity Behaviors Do Not Readily Explain Why Smokers Gain Weight After Cessation: A Cohort Study in Young Adults. *Nicotine Tob. Res.* **2017**, *19*, 357–366, doi:10.1093/ntr/ntw196.
97. Wahlqvist, M.L.; Kouris-blazos, A.; Wattanapenpaiboon, N. The significance of eating patterns: an elderly Greek case study. *Appetite* **1999**, *32*, 23–32, doi:10.1006/appe.1998.0192.
98. Wardle, J.; Griffith, J.; Johnson, F.; Rapoport, L. Intentional weight control and food choice habits in a national representative sample of adults in the UK. *Int. J. Obes. Relat. Metab. Disord.* **2000**, *24*, 534–540.
99. Alacid, F.; Vaquero-Cristobal, R.; Sanchez-Pato, A.; Muyor, J.M.; Lopez-Minarro, P.A. Habit based consumptions in the mediterranean diet and the relationship with anthropometric parameters in young female kayakers. *Nutr. Hosp.* **2014**, *29*, 121–127, doi:10.3305/nh.2014.29.1.6995.
100. Bion, F.M.; Chagas, M H de Castro; Muniz, G.d.S.; de Sousa, L G Oliveira. Nutritional status, anthropometrical measurements, socio-economic status, and physical activity in Brazilian university students. *Nutr. Hosp.* **2008**, *23*, 234–241.
101. Chiu, H.-C.; Wu, C.-F.; Lee, H.-L.; Chen, M.-Y. Health related behaviors and associated factors among visitors to a health promotion center. *Hu Li Za Zhi* **2008**, *55*, 39–48.
102. Duran Aguero, S.; Fernandez Godoy, E.; Fuentes Fuentes, J.; Hidalgo Fernandez, A.; Quintana Munoz, C.; Yunge Hidalgo, W.; Fehrman Rosas, P.; Delgado Sanchez, C. FOOD PATTERNS ASSOCIATED WITH A HEALTY BODY WEIGHT IN CHILEAN STUDENTS OF NUTRITION AND DIETETICS. *Nutr. Hosp.* **2015**, *32*, 1780–1785, doi:10.3305/nh.2015.32.4.9515.
103. Egeda Manzanera, J.M.; Rodrigo Vega, M. Adherence to the Mediterranean diet of future teachers. *Nutr. Hosp.* **2014**, *30*, 343–350, doi:10.3305/nh.2014.30.2.7585.
104. Fukunaga, I.; Jitsunari, F.; Takeda, N.; Asakawa, F.; Maruyama, Y. A study of health behavior of the elderly without occupation--correlation between participation in health examinations and health behavior. *Nihon Eiseigaku Zasshi* **1997**, *52*, 490–503.
105. Hage, C.N.; Sayegh, J.; Rizk, G.A. Health habits and vaccination status of Lebanese residents: are future doctors applying the rules of prevention? *J. Med. Liban.* **2010**, *58*, 91–96.
106. Hu, C.H.; Zhang, M.; Zhang, X.; Zhao, Z.P.; Huang, Z.J.; Li, C.; Wang, X.; Guan, Y.Q.; Wang, L.M. Relationship between eating behavior and obesity among Chinese adults. *Zhonghua Liu Xing Bing Xue Za Zhi* **2020**, *41*, 1296–1302, doi:10.3760/cma.j.cn112338-20191225-00915.
107. Kahleova, H.; Lloren, J.I.; Mashchak, A.; Hill, M.; Fraser, G. Frequency and timing of meals and changes in body mass index: Analysis of the data from the Adventist Health Study-2. *Vnitr. Lek.* **2016**, *62*, S15-20.

108. Kasamaki, J.; Miyanishi, K.; Kasahara, Y.; Matsumoto, H.; Nishida, J.; Shibukura, T. Factors Related to Nutritional Intake in Students Attending Universities, Colleges, and Vocational Schools: Focus on Gender and Household Living Arrangement. *Nihon Eiseigaku Zasshi* **2018**, *73*, 395–412, doi:10.1265/jjh.73.395.
109. Morel, Y. Dietary approach to obesity. *Praxis (Bern 1994)* **1996**, *85*, 1584–1588.
110. Nakano, K.; Yabe, J.; Yasumura, S. Health practice and total mortality among middle-aged and elderly residents in Sukagawa, Japan. *Nihon. Koshu Eisei Zasshi.* **2006**, *53*, 329–337.
111. Osako, M.; Takayama, T.; Kira, S. Dietary habits, attitudes toward weight control, and subjective symptoms of fatigue in young women in Japan. *Nihon. Koshu Eisei Zasshi.* **2005**, *52*, 387–398.
112. Ratner, R.G.; Hernandez, P.J.; Martel, J.A.; Atalah, E.S. Food quality and nutritional status in university students of eleven Chilean regions. *Rev. Med. Chil.* **2012**, *140*, 1571–1579, doi:10.4067/S0034-98872012001200008.
113. Redondo, M.R.; Ortega, R.M.; Lopez-Sobaler, A.M.; Quintas, M.E.; Andres, P. Food, energy and nutrient intake at breakfast in a group of elderly persons. Most common problems and differences related to body mass index. *Arch. Latinoam. Nutr.* **1996**, *46*, 275–281.
114. Rodriguez, F.; Palma, X.; Romo, A.; Escobar, D.; Aragu, B.; Espinoza, L.; McMillan, N.; Galvez, J. Eating habits, physical activity and socioeconomic level in university students of Chile. *Nutr. Hosp.* **2013**, *28*, 447–455, doi:10.3305/nh.2013.28.2.6230.
115. Soga, Y.; Shirai, C.; Ijichi, A. Association between daily lifestyle and the risk of metabolic syndrome among young adults in Japan. An analysis of Kobe city young adult health examination data. *Nihon. Koshu Eisei Zasshi.* **2013**, *60*, 98–106.
116. Spagnoli, T.D.; Bianco, L. Breakfast habits in moderately obese adults and its effect on daily energy and nutrient intake, on alcohol consumption and on various clinical and anthropometric parameters. *Minerva Gastroenterol. Dietol.* **1995**, *41*, 237–245.
117. Sugisawa, A.; Sugisawa, H.; Shibata, H. Impact of health practices on changes in the physical and mental well-being of older adults. *Nihon. Koshu Eisei Zasshi.* **1998**, *45*, 104–111.
118. Teixeira, L.K.; Narloch Rizzo Hahn, L.R. Association between breakfast skipping and nutritional status in employees of a technology company in Joinville. *RBONE-REVISTA BRASILEIRA DE OBESIDADE NUTRICAO E EMAGRECIMENTO* **2018**, *12*, 431–438.
119. Yoo, K.-B.; Suh, H.-J.; Lee, M.; Kim, J.-H.; Kwon, J.A.; Park, E.-C. Breakfast eating patterns and the metabolic syndrome: the Korea National Health and Nutrition Examination Survey (KNHANES) 2007-2009. *Asia Pac. J. Clin. Nutr.* **2014**, *23*, 128–137, doi:10.6133/apjcn.2014.23.1.08.
120. Zhang, P.-H.; Jiao, S.-F.; Zhou, Y.; Wang, H.-B.; Wu, F.; Jiang, Y.; Liu, Z.-J. Study on chronic disease related behavior and lifestyle in adults in Beijing, 2005. *Zhonghua Liu Xing Bing Xue Za Zhi* **2007**, *28*, 1162–1166.
121. Adeoso, A.; Atinmo, T. Comparative analysis of dietary pattern, anthropometry and serum ascorbate status of persons living with or without non-Hodgkin's lymphoma. *J. Public Health Afr.* **2018**, *9*, 768, doi:10.4081/jphia.2018.768.
122. Alzamil, H.A.; Alhakbany, M.A.; Alfadda, N.A.; Almusallam, S.M.; Al-Hazzaa, H.M. A Profile of Physical Activity, Sedentary Behaviors, Sleep, and Dietary Habits of Saudi College Female Students. *J. Family Community Med.* **2019**, *26*, 1–8, doi:10.4103/jfcm.JFCM_58_18.
123. Cahill, L.E.; Chiuve, S.E.; Mekary, R.A.; Jensen, M.K.; Flint, A.J.; Hu, F.B.; Rimm, E.B. Prospective study of breakfast eating and incident coronary heart disease in a cohort of male US health professionals. *Circulation* **2013**, *128*, 337–343, doi:10.1161/CIRCULATIONAHA.113.001474.
124. Cakar, U.; Sobajic, S.; Vidovic, B.; Djordjevic, B. Nutritional and lifestyle habits of European pharmacy undergraduate students. *PROGRESS IN NUTRITION* **2018**, *20*, 38–45, doi:10.23751/pn.v20i1.5435.
125. Clarke, P.J.; O'Malley, P.M.; Johnston, L.D.; Schulenberg, J.E.; Lantz, P. Differential trends in weight-related health behaviors among American young adults by gender, race/ethnicity, and socioeconomic status: 1984-2006. *Am. J. Public Health* **2009**, *99*, 1893–1901, doi:10.2105/AJPH.2008.141317.
126. Fransen, H.P.; Boer, J.M.A.; Beulens, J.W.J.; Wit, G.A. de; Bueno-de-Mesquita, H.B.; Hoekstra, J.; May, A.M.; Peeters, P.H.M. Associations between lifestyle factors and an unhealthy diet. *Eur. J. Public Health* **2017**, *27*, 274–278, doi:10.1093/eurpub/ckw190.
127. Fujii, H.; Nakano, T.; Muto, T.; Aikawa, K. Skipping Breakfast is Associated with Poor Vegetable Intake Among College Students in Japan. *Dokkyo Journal of Medical Sciences* **2010**, *37*, 47–54.
128. Hojat, M.; Jahromi, M.K.; Koshkaki, S.R.; Rahmanian, M. Comparison of risk factors of cardiovascular diseases in male and female nurses. *J. Educ. Health Promot.* **2019**, *8*, 19, doi:10.4103/jehp.jehp_221_18.
129. Kim, S.; Ko, Y.; Yi, G. Role of social determinants and lifestyle on women's metabolic risk during the perimenopausal transition: results from a cohort study. *Menopause* **2016**, *23*, 403–409, doi:10.1097/GME.0000000000000544.
130. Likus, W.; Milka, D.; Bajor, G.; Jachacz-Lopata, M.; Dorzak, B. Dietary habits and physical activity in students from the Medical University of Silesia in Poland. *Rocz. Panstw. Zakl. Hig.* **2013**, *64*, 317–324.
131. Macera, C.A.; Pate, R.R.; Davis, D.R. Runners' health habits, 1985--"the Alameda 7" revisited. *Public Health Rep.* **1989**, *104*, 341–349.

132. Nishiyama, M.; Muto, T.; Minakawa, T.; Shibata, T. The Combined Unhealthy Behaviors of Breakfast Skipping and Smoking Are Associated with the Prevalence of Diabetes Mellitus. *Tohoku J. Exp. Med.* **2009**, *218*, 259–264, doi:10.1620/tjem.218.259.
133. Omage, K.; Omuemu, V.O. Assessment of dietary pattern and nutritional status of undergraduate students in a private university in southern Nigeria. *FOOD SCIENCE & NUTRITION* **2018**, *6*, 1890–1897, doi:10.1002/fsn3.759.
134. Paulitsch, R.G.; Dumith, S.C.; Susin, L.R.O. Simultaneity of behavioral risk factors for cardiovascular disease in university students. *Rev. Bras. Epidemiol.* **2017**, *20*, 624–635, doi:10.1590/1980-5497201700040006.
135. Poscia, A.; Teleman, A.A.; Azzolini, E.; Waure, C. de; Maged, D.; Virdis, A.; Ricciardi, W.; Di Pietro, M.L. Eating episode frequency and fruit and vegetable consumption among Italian university students. *Ann. Ist. Super. Sanita* **2017**, *53*, 199–204, doi:10.4415/ANN_17_03_04.
136. Schoenborn, C.A. Health habits of U.S. adults, 1985: the "Alameda 7" revisited. *Public Health Rep.* **1986**, *101*, 571–580.
137. Schusdziarra, V.; Hausmann, M.; Wittke, C.; Mittermeier, J.; Kellner, M.; Naumann, A.; Wagenpfeil, S.; Erdmann, J. Impact of breakfast on daily energy intake--an analysis of absolute versus relative breakfast calories. *Nutr. J.* **2011**, *10*, 5, doi:10.1186/1475-2891-10-5.
138. Segovia, J.; Bartlett, R.F.; Edwards, A.C. The association between self-assessed health status and individual health practices. *Can. J. Public Health* **1989**, *80*, 32–37.
139. Suliga, E.; Wronka, I.; Pawlińska-Chmara, R. Nutritional habits of female university students in relation to selfperception of body. *Biomedical Human Kinetics* **2012**, *4*, doi:10.2478/v10101-012-0018-9.
140. Williams, P. Breakfast and the diets of Australian adults: an analysis of data from the 1995 National Nutrition Survey. *Int. J. Food Sci. Nutr.* **2005**, *56*, 65–79, doi:10.1080/09637480500082108.
141. Yahia, N.; Achkar, A.; Abdallah, A.; Rizk, S. Eating habits and obesity among Lebanese university students. *Nutr. J.* **2008**, *7*, 32, doi:10.1186/1475-2891-7-32.
142. Almoosawi, S.; Prynne, C.J.; Hardy, R.; Stephen, A.M. Time-of-day and nutrient composition of eating occasions: prospective association with the metabolic syndrome in the 1946 British birth cohort. *Int. J. Obes. (Lond)* **2013**, *37*, 725–731, doi:10.1038/ijo.2012.103.
143. Barr, S.I.; DiFrancesco, L.; Fulgoni, V.L. Consumption of breakfast and the type of breakfast consumed are positively associated with nutrient intakes and adequacy of Canadian adults. *J. Nutr.* **2013**, *143*, 86–92, doi:10.3945/jn.112.167098.
144. Breslow, L.; Breslow, N. Health practices and disability: some evidence from Alameda County. *Prev. Med.* **1993**, *22*, 86–95, doi:10.1006/pmed.1993.1006.
145. Castro, J.M. de. When, how much and what foods are eaten are related to total daily food intake. *Br. J. Nutr.* **2009**, *102*, 1228–1237, doi:10.1017/S0007114509371640.
146. Cebirbay, M.A.; Aktas, N.; Calderoni, M. Determination of breakfast habits and knowledge of foreign undergraduates studying at Selcuk University in Turkey. *PROGRESS IN NUTRITION* **2011**, *13*, 276–285.
147. Colic Baric, I.; Satalic, Z.; Lukesic, Z. Nutritive value of meals, dietary habits and nutritive status in Croatian university students according to gender. *Int. J. Food Sci. Nutr.* **2003**, *54*, 473–484, doi:10.1080/09637480310001622332.
148. Gutierrez-Salmean, G.; Meaney, A.; Ocharan, M.E.; Araujo, J.M.; Ramirez-Sanchez, I.; Olivares-Corichi, I.M.; Garcia-Sanchez, R.; Castillo, G.; Mendez-Bolaina, E.; Meaney, E.; et al. Anthropometric traits, blood pressure, and dietary and physical exercise habits in health sciences students; the obesity observatory project. *Nutr. Hosp.* **2013**, *28*, 194–201, doi:10.3305/nh.2013.28.1.6185.
149. Nola, I.A.; Jelinic, J.D.; Matanic, D.; Pucarin-Cvetkovic, J.; Bergman Markovic, B.; Senta, A. Differences in eating and lifestyle habits between first- and sixth-year medical students from Zagreb. *Coll. Antropol.* **2010**, *34*, 1289–1294.
150. Nti, C.A. Household dietary practices and family nutritional status in rural Ghana. *Nutr. Res. Pract.* **2008**, *2*, 35–40, doi:10.4162/nrp.2008.2.1.35.
151. Reicks, M.; Degeneffe, D.; Rendahl, A.; Smith Edge, M.; Burns, K.; O'Meara, B.; Blevins, G. Associations between eating occasion characteristics and age, gender, presence of children and BMI among U.S. adults. *J. Am. Coll. Nutr.* **2014**, *33*, 315–327, doi:10.1080/07315724.2014.887485.
152. Satalic, Z.; Baric, I.C.; Keser, I. Diet quality in Croatian university students: energy, macronutrient and micronutrient intakes according to gender. *Int. J. Food Sci. Nutr.* **2007**, *58*, 398–410, doi:10.1080/09637480701252393.
153. Setayeshgar, S.; Whiting, S.J.; Pahwa, P.; Vatanparast, H. Predicted 10-year risk of cardiovascular disease among Canadian adults using modified Framingham Risk Score in association with dietary intake. *Appl. Physiol. Nutr. Metab.* **2015**, *40*, 1068–1074, doi:10.1139/apnm-2015-0074.
154. Sun, J.; Yi, H.; Liu, Z.; Wu, Y.; Bian, J.; Wu, Y.; Eshita, Y.; Li, G.; Zhang, Q.; Yang, Y. Factors associated with skipping breakfast among Inner Mongolia Medical students in China. *BMC Public Health* **2013**, *13*, doi:10.1186/1471-2458-13-42.

155. Trivedi, T.; Liu, J.; Probst, J.C.; Martin, A.B. The metabolic syndrome: are rural residents at increased risk? *J. Rural Health* **2013**, *29*, 188–197, doi:10.1111/j.1748-0361.2012.00422.x.
156. Vogel, S.W.N.; Bijlenga, D.; Tanke, M.; Bron, T.I.; van der Heijden, Kristiaan B; Swaab, H.; Beekman, A.T.F.; Kooij, J.J.S. Circadian rhythm disruption as a link between Attention-Deficit/Hyperactivity Disorder and obesity? *J. Psychosom. Res.* **2015**, *79*, 443–450, doi:10.1016/j.jpsychores.2015.10.002.
157. Chen, M.-Y.; Liao, J.C. Relationship between attendance at breakfast and school achievement among nursing students. *J. Nurs. Res.* **2002**, *10*, 15–21.
158. Christoforidis, A.; Batzios, S.; Sidiropoulos, H.; Provatidou, M.; Cassimos, D. The profile of the Greek 'XXL' family. *Public Health Nutr.* **2011**, *14*, 1851–1857, doi:10.1017/S1368980011000929.
159. Goff, L.M.; Huang, P.; Silva, M.J.; Bordoli, C.; Enayat, E.Z.; Molaodi, O.R.; Cassidy, A.; Maynard, M.; Harding, S. Associations of dietary intake with cardiometabolic risk in a multi-ethnic cohort: a longitudinal analysis of the Determinants of Adolescence, now young Adults, Social well-being and Health (DASH) study. *Br. J. Nutr.* **2019**, *121*, 1069–1079, doi:10.1017/S0007114519000291.
160. Laska, M.N.; Murray, D.M.; Lytle, L.A.; Harnack, L.J. Longitudinal associations between key dietary behaviors and weight gain over time: transitions through the adolescent years. *Obesity (Silver Spring)* **2012**, *20*, 118–125, doi:10.1038/oby.2011.179.
161. Merten, M.J.; Williams, A.L.; Shriver, L.H. Breakfast consumption in adolescence and young adulthood: parental presence, community context, and obesity. *J. Am. Diet. Assoc.* **2009**, *109*, 1384–1391, doi:10.1016/j.jada.2009.05.008.
162. Niemeier, H.M.; Raynor, H.A.; Lloyd-Richardson, E.E.; Rogers, M.L.; Wing, R.R. Fast food consumption and breakfast skipping: predictors of weight gain from adolescence to adulthood in a nationally representative sample. *J. Adolesc. Health* **2006**, *39*, 842–849, doi:10.1016/j.jadohealth.2006.07.001.
163. Olatona, F.A.; Onabanjo, O.O.; Ugbaja, R.N.; Nnoaham, K.E.; Adelekan, D.A. Dietary habits and metabolic risk factors for non-communicable diseases in a university undergraduate population. *J. Health Popul. Nutr.* **2018**, *37*, 21, doi:10.1186/s41043-018-0152-2.
164. Quick, V.; Wall, M.; Larson, N.; Haines, J.; Neumark-Sztainer, D. Personal, behavioral and socio-environmental predictors of overweight incidence in young adults: 10-yr longitudinal findings. *Int. J. Behav. Nutr. Phys. Act.* **2013**, *10*, 37, doi:10.1186/1479-5868-10-37.
165. Serra-Majem, L.; Aranceta Bartrina, J.; Pérez-Rodrigo, C.; Ribas-Barba, L.; Delgado-Rubio, A. Prevalence and deteminants of obesity in Spanish children and young people. *Br. J. Nutr.* **2006**, *96 Suppl 1*, S67-72.
166. Goyal, R.; Julka, S. Impact of breakfast skipping on the health status of the population. *Indian J. Endocrinol. Metab.* **2014**, *18*, 683–687, doi:10.4103/2230-8210.139233.
167. Sakurai, M.; Yoshita, K.; Nakamura, K.; Miura, K.; Takamura, T.; Nagasawa, S.Y.; Morikawa, Y.; Kido, T.; Naruse, Y.; Nogawa, K.; et al. Skipping breakfast and 5-year changes in body mass index and waist circumference in Japanese men and women. *Obes. Sci. Pract.* **2017**, *3*, 162–170, doi:10.1002/osp4.106.
168. Wengreen, H.J.; Moncur, C. Change in diet, physical activity, and body weight among young-adults during the transition from high school to college. *Nutr. J.* **2009**, *8*, 32, doi:10.1186/1475-2891-8-32.
169. Dundes, L. Fleshing out the role of skipping breakfast in weight control. *AGRO FOOD INDUSTRY HI-TECH* **2008**, *19*, 26–27.
170. Lauque, S.; Nourashemi, F.; Soleilhavoup, C.; Guyonnet, S.; Bertiere, M.C.; Sachet, P.; Vellas, B.; Albarede, J.L. A prospective study of changes on nutritional patterns 6 months before and 18 months after retirement. *J. Nutr. Health Aging* **1998**, *2*, 88–91.
171. Goto, M.; Kiyohara, K.; Kawamura, T. Lifestyle risk factors for overweight in Japanese male college students. *Public Health Nutr.* **2010**, *13*, 1575–1580, doi:10.1017/S1368980009992813.
172. Guinter, M.A.; Park, Y.-M.; Steck, S.E.; Sandler, D.P. Day-to-day regularity in breakfast consumption is associated with weight status in a prospective cohort of women. *Int. J. Obes. (Lond)* **2020**, *44*, 186–194, doi:10.1038/s41366-019-0356-6.
173. Hurst, Y.; Fukuda, H. Effects of changes in eating speed on obesity in patients with diabetes: a secondary analysis of longitudinal health check-up data. *BMJ Open* **2018**, *8*, e019589, doi:10.1136/bmjopen-2017-019589.
174. Kahleova, H.; Lloren, J.I.; Mashchak, A.; Hill, M.; Fraser, G.E. Meal Frequency and Timing Are Associated with Changes in Body Mass Index in Adventist Health Study 2. *J. Nutr.* **2017**, *147*, 1722–1728, doi:10.3945/jn.116.244749.
175. Kito, K.; Kuriyama, A.; Takahashi, Y.; Nakayama, T. Impacts of skipping breakfast and late dinner on the incidence of being overweight: a 3-year retrospective cohort study of men aged 20-49 years. *J. Hum. Nutr. Diet.* **2019**, doi:10.1111/jhn.12640.
176. Nooyens, A.C.J.; Visscher, T.L.S.; Schuit, A.J.; van Rossum, Caroline T M; Verschuren, W.M.M.; van Mechelen, W.; Seidell, J.C. Effects of retirement on lifestyle in relation to changes in weight and waist circumference in Dutch men: a prospective study. *Public Health Nutr.* **2005**, *8*, 1266–1274.
177. Odegaard, A.O.; Jacobs, D.R., JR; Steffen, L.M.; van Horn, L.; Ludwig, D.S.; Pereira, M.A. Breakfast frequency and development of metabolic risk. *Diabetes Care* **2013**, *36*, 3100–3106, doi:10.2337/dc13-0316.

178. Smith, K.J.; Gall, S.L.; McNaughton, S.A.; Cleland, V.J.; Otahal, P.; Dwyer, T.; Venn, A.J. Lifestyle behaviours associated with 5-year weight gain in a prospective cohort of Australian adults aged 26-36 years at baseline. *BMC Public Health* **2017**, *17*, 54, doi:10.1186/s12889-016-3931-y.
179. van der Heijden, A.A.W.A.; Hu, F.B.; Rimm, E.B.; van Dam, R.M. A prospective study of breakfast consumption and weight gain among U.S. men. *Obesity (Silver Spring)* **2007**, *15*, 2463–2469, doi:10.1038/oby.2007.292.
